# Augmented Reality in Navigated Surgery: A Systematic Review of Clinical Accuracy and System Performance

**DOI:** 10.1016/j.mcpdig.2026.100358

**Published:** 2026-04-04

**Authors:** Noa Nicolai, Sander J.C. Tabernée Heijtmeijer, Mohamed Benmahdjoub, Joep Kraeima, Sarina E.C. Pichardo, Max J.H. Witjes, Peter A.J. Pijpker, Joël Kortes, Nard G. Janssen, Jan Eelco Bergsma

**Affiliations:** a 3D Lab, University Medical Center Utrecht, Netherlands; b Department of Oral and Maxillofacial Surgery, University Medical Center Utrecht, Netherlands; c 3D Lab, University Medical Center Groningen, University of Groningen, Netherlands; d Department of Oral and Maxillofacial Surgery, University Medical Center Groningen, University of Groningen, Netherlands; e Department of Oral and Maxillofacial Surgery, Erasmus MC, University Medical Center Rotterdam, Netherlands; f Biomedical Imaging Group Rotterdam, Department of Radiology and Nuclear Medicine, Erasmus MC, University Medical Center Rotterdam, Netherlands

## Abstract

**Objective:**

To evaluate the current state of clinically tested augmented reality and mixed reality, hereafter referred to as AR, navigation systems, with a focus on accuracy, usability, and factors influencing clinical implementation.

**Methods:**

A systematic search was conducted in Embase, PubMed, Scopus, and Web of Science Core Collection including studies published between January 1, 2018, and July 14, 2025. Only clinical studies involving patients were included. Data extraction covered study characteristics, surgical specialty, AR system type, reported accuracy, usability assessments, and implementation-related factors.

**Results:**

Of 1956 screened records, 61 studies met the inclusion criteria. The applications spanned oral and maxillofacial surgery (k=17), neurosurgery (k=12), spinal surgery (k=11), and orthopedics (k=10), among others. Reported accuracy metrics varied substantially across studies. Moreover, AR navigation often reduced radiation exposure during surgery and sometimes shortened operative time, although timing effects varied by specialty. Usability was rarely measured with standardized tools and was mostly described qualitatively. Common limitations were limited accuracy, ergonomic issues, and workflow-integration challenges. Grading of Recommendations Assessment, Development, and Evaluation ratings were generally low or very low owing to small samples, heterogeneous methods, and risk of bias.

**Conclusion:**

Although AR navigation demonstrates encouraging technical performance and potential reductions in radiation exposure, the underlying evidence is predominantly of very low certainty, and any impression of pooled robustness should be avoided. Furthermore, clinical integration is hindered by technical, ergonomic, and workflow-related barriers. Future work should incorporate robust methodological designs aimed at improving registration accuracy, overcoming hardware shortcomings, and systematically evaluating usability through validated, standardized tools.

## Graphical abstract

**Figure:**
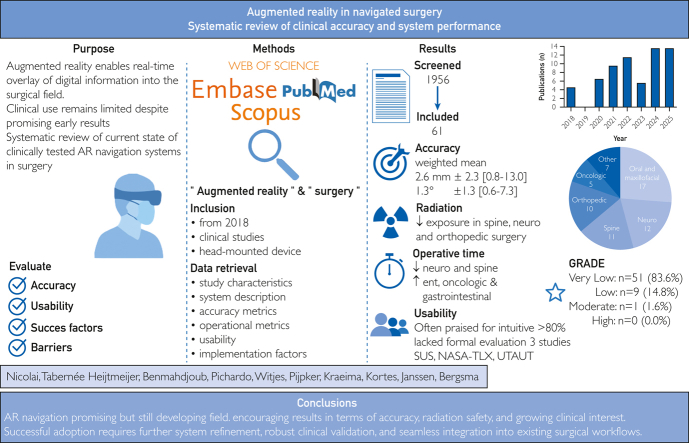

**Article Highlights**

• First review of only clinically tested head-mounted display–augmented reality navigation systems using a structured taxonomy.
• Workflow, ergonomics, and usability remain key adoption challenges.
• Grading of Recommendations Assessment, Development, and Evaluation assessments indicated predominantly low or very low certainty of evidence across outcomes. This indicates the need for future research to adopt more standardized evaluation methods.
• On the basis of the findings, a minimum reporting set for future augmented reality surgical navigation studies is suggested.

Virtual surgical planning (VSP) has become an important tool for supporting surgeons in their preoperative preparation and enables the creation of digital simulations and patient-specific 3-dimensional (3D) anatomical models in either virtual or physical, 3D-printed models. To ensure the preoperative plan is accurately transferred to the operating room, additional technologies, such as patient specific surgical guides or navigation systems, can be used.

Surgical navigation systems can accurately track the spatial location of both instruments and the patient and correlate with the relative positions in the preoperative images. The instruments’ position is projected onto the preoperative imaging data, containing the VSP. The locations are continuously updated and shown on a monitor. Therefore, real-time tracking of the instruments is possible, and the surgeon can be guided throughout the procedure. Commonly, optical tracking systems are used to track the patient and instruments. Surgical navigation systems are widely used in various surgical domains to assist the surgeon during complex procedures. Image-guided surgery can improve surgical outcomes and contribute to faster, safer, and more effective surgical procedures.^1^ One major drawback of image-guided surgery is the need to pay attention to the patient and the monitor of the navigational system. The attention shift negatively impacts the cognitive and motor tasks of the surgeon, disrupts the continuity of the surgery, and decreases the situational awareness of the surgical field.^2^, ^3^, ^4^ Furthermore, the 3D VSP is shown on a 2D display and is not aligned with the viewpoint of the surgeon, which is another factor for a negative impact on cognitive load.^1^^,^^5^

Extended reality technologies have gained increasing attention in surgical practice over the past decade. By overlaying digital information onto the real surgical field, these systems have the potential to enhance anatomical orientation, improve intraoperative navigation, and reduce the cognitive load of surgeons.^4^^,^^6^^,^^7^ Unlike virtual reality, which immerses users in a fully digital environment, augmented reality (AR) keeps the real world visible while superimposing computer-generated information upon it.^4^^,^^7^ Mixed reality goes a step further by enabling interaction and integration between real-world elements and virtual objects. Although AR and mixed reality are distinct, the terms are often used interchangeably. For our study, therefore, both terms are grouped under the AR. AR is a highly promising technology for navigation in surgical applications, because it can guide the surgeon or give additional information without having to switch attention between the patient and the navigation monitor.^1^^,^^4^^,^^6^^,^^8^^,^^9^ Head-mounted display (HMD) AR devices enable the overlay of virtual models directly onto real surgical fields, assisting surgeons in complex procedures and potentially improving patient outcomes.^4^^,^^10^

Despite promising early results, clinical adoption of AR systems remains limited.^11^, ^12^, ^13^ One of the main challenges lies in translating experimental or technical feasibility into robust, reliable, and user-friendly solutions that can be seamlessly integrated into daily clinical workflows. Hospitals considering implementation face questions regarding usability, accuracy, workflow compatibility, and cost-effectiveness. Moreover, requirements may vary across surgical specialties, raising the issue of whether a single flexible platform can meet the needs of different departments or whether specialized systems are required. Although several clinical studies have already been conducted, the key success factors and remaining challenges for clinical implementation have not yet been systematically mapped.

This systematic review aims to fill that gap by evaluating the current state of clinically tested HMD-AR navigation systems across different surgical specialties. The available literature was synthesized to assess their clinical accuracy and usability and identify factors that facilitate or hinder successful adoption.

Primary outcomes include the accuracy of AR systems and their usability in clinical workflows, including success factors and barriers to implementation. Secondary outcomes include technical system characteristics (hardware, tracking, and registration methods), comparative performance against conventional techniques, required preparation time, and their impact on operative time and radiation exposure (Graphical Abstract).

## Methods

### Eligibility

This study was conducted using the Preferred Reporting Items for Systematic Reviews and Meta-analyses (PRISMA) 2020 statement as a guideline. Eligibility criteria were defined to focus specifically on clinically deployed, HMD-AR navigation systems, representing a subset of AR applications that differ substantially from endoscopic, laparoscopic, robotic, or microscopic AR use cases. These other categories were excluded because they use fundamentally different display paradigms, workflows, and system architectures.

### Literature Search

Literature searches were conducted in the following databases: Embase, PubMed, Scopus, and Web of Science Core Collection. Key search terms included the following—*augmented reality*, *extended reality*, *surgery*, *computer-assisted surgery*, *operating theater*, and synonyms, plurals, and different spellings of these terms. The query strings can be found in Supplemental Appendix 1 (available online at https://www.mcpdigitalhealth.org/). Publications from January 1, 2018, to July 14, 2025, were searched. The 2018-2025 window reflects the period in which clinically usable HMD-based AR navigation systems became available (eg, second-generation Microsoft HoloLens). Pre-2018 work was largely prototype-based or used outdated hardware and would not be representative of modern clinical systems. The English-language restriction was applied to ensure reliable assessment of methodological quality and technical descriptions, acknowledging that this may introduce language bias. Duplicates were removed before screening.

### Study Selection

The titles and abstracts of all the studies resulting from the search were screened by 2 authors independently (N.N. and S.J.C.T.-H.). Studies were excluded based on the following exclusion criteria:

1. Articles published before 2018;
2. Non-scientific publications;
3. Conference papers or book chapters;
4. Studies limited to phantom models, cadaveric experiments, or purely technical feasibility assessments without patient involvement;
5. Articles describing virtual reality systems, non-HMD systems (eg, handheld devices or projection based AR), or systems that do not meet the definition of AR/mixed reality as outlined in the introduction;
6. Articles describing AR systems solely for training purposes;
7. Articles where AR is used for telementoring, microscopic surgery, laparoscopic/endoscopic surgery, or robotic surgery;
8. Articles describing AR systems not applicable to surgical procedures or where the use of the AR system in a surgical setting is not described;
9. Studies focusing only on a specific component of the surgical navigation system rather than the system as a whole.

The full texts of the remaining articles were assessed by the same authors to determine eligibility. Articles were included if they met all of the following criteria:

1. The article describes a new or existing HMD-AR systems that translates the 3D VSP into the operating room and is used to guide the surgeon during the procedure;
2. The study reports in vivo clinical research involving human subjects undergoing surgical procedures.

Any disagreements regarding the relevance of references were resolved through discussion and consensus between the 2 authors.

### Quality Assessment

Quality assessment was performed based on the GRADE (Grading of Recommendations Assessment, Development, and Evaluation) methodology, as outlined by Prasad.^14^ The initial GRADE rating was determined based on the study design. Two reviewers (N.N. and S.J.C.T.-H.) independently assessed each study for potential downgrading or upgrading based on predefined GRADE domains. The final rating was determined by combining the initial rating with the cumulative impact of downgrading and/or upgrading across domains. Conflicting results between the 2 reviewers were resolved through discussion to reach consensus. This resulted in one of the following GRADE certainty level and were interpreted as defined by Prasad and as follows:

• High certainty—high level of confidence in the estimate of the effect. Further research is unlikely to significantly change the confidence.
• Moderate certainty—the available evidence supports the conclusion. However, further research may impact the confidence in the estimate.
• Low certainty—limited evidence is available and the true effect may differ significantly from the estimate.
• Very low certainty—insufficient evidence is available to support any firm conclusions.^14^

### Data Retrieval

The following parameters were extracted from the included studies:

• Study characteristics: publication year, authorship, study design, sample size, and medical specialty.
• System description: categorized according to the taxonomy proposed by Gsaxner et al,^15^ including hardware and software specifications.
• Accuracy metrics: reported target accuracy, measurement methods, and positional/angular accuracy outcomes.
• Usability: user-friendliness and ease of integration into the surgical workflow.
• Implementation factors: facilitators and barriers to clinical adoption.
• Operational metrics: preparation time, surgical duration, and radiation exposure.

### Data Analysis

A narrative synthesis was performed to summarize findings across studies and to provide an overview of the clinical application of HMD-AR systems in surgical procedures. Data were categorized based on surgical specialty and system type, following the taxonomy proposed by Gsaxner et al.^15^ Where homogeneous data were available, pooling was conducted for accuracy metrics, preparation and surgical time, and radiation exposure. Qualitative data on usability and implementation factors were synthesized thematically to identify recurring success and limiting factors across studies. Owing to heterogeneity in study designs and outcome measures, a meta-analysis was not conducted.

## Results

### Study Selection

**Figure 1:**
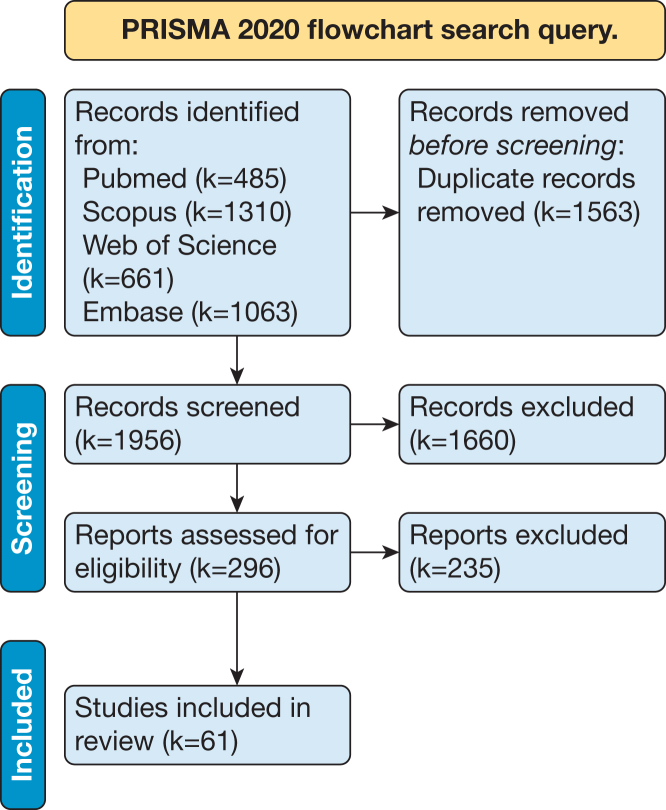
Preferred Reporting Items for Systematic Reviews and Meta-analyses 2020 flowchart search query. k, number of articles.

The search strategy was performed on July 14, 2025, and resulted in a total of 3519 articles. All duplicates were removed, and a total of 1956 articles remained for title and abstract screening (Figure 1). After application of the selection criteria, k=61 articles were included (k=number of articles). Figure 1 shows the flow diagram including the study selection results.

### Study Characteristics

**Figure 2:**
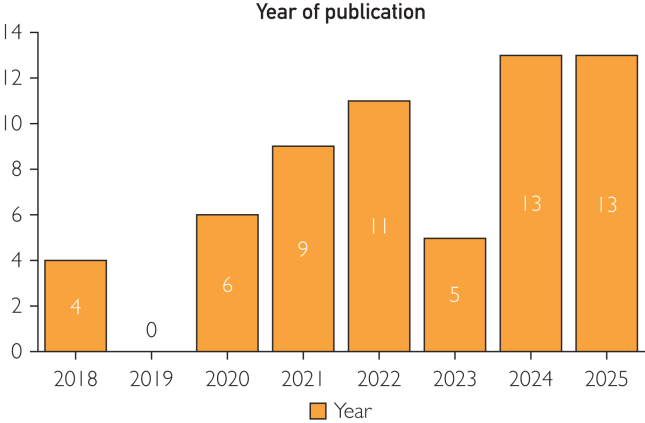
Overview of publication year of the included studies.

**Figure 3:**
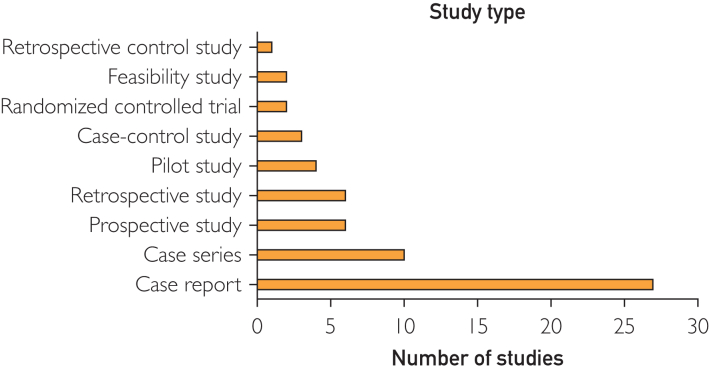
Bar chart of the study type of the included articles.

Study characteristics are summarized in Table 1.^16^, ^17^, ^18^, ^19^, ^20^, ^21^, ^22^, ^23^, ^24^, ^25^, ^26^, ^27^, ^28^, ^29^, ^30^, ^31^, ^32^, ^33^, ^34^, ^35^, ^36^, ^37^, ^38^, ^39^, ^40^, ^41^, ^42^, ^43^, ^44^, ^45^, ^46^, ^47^, ^48^, ^49^, ^50^, ^51^, ^52^, ^53^, ^54^, ^55^, ^56^, ^57^, ^58^, ^59^, ^60^, ^61^, ^62^, ^63^, ^64^, ^65^, ^66^, ^67^, ^68^, ^69^, ^70^, ^71^, ^72^, ^73^, ^74^, ^75^, ^76^ The studies were published between 2018 and 2025. Figure 2 shows an overview of the publication year of the included articles. Most of the studies were case reports (k=27) and case series (k=10). Only 2 studies were randomized controlled trials. Figure 3 shows an overview of the study types of the included articles. The entire study population included n_tot_=1547 patients of which n_AR_=1033 were evaluated in the AR group (n_tot_=total number of patients; n_AR_=number of patients in the AR group).

**Table 1:** Study Characteristics

Reference, year	Study type	Sample size (total, n_tot_)	Sample size (AR, n_AR_)	Medical specialty
Incekara et al,^16^ 2018	Prospective study	25	25	Neurosurgery
Zhu et al,^17^ 2018	Retrospective study	93	31	Oral and maxillofacial surgery
Maruyama et al,^18^ 2018	Case series	2	2	Neurosurgery
Gregory et al,^19^ 2018	Case report	1	1	Orthopedic surgery
Gu et al,^20^ 2020	Case series	50	25	Spine surgery
Yao et al,^21^ 2020	Prospective study	5	5	Neurosurgery
Scherl et al,^22^ 2020	Feasibility study	7	7	Ear, nose, and throat surgery
Molina et al,^23^ 2021	Case report	1	1	Spine surgery
Sun et al,^24^ 2020	Case report	1	1	Oral and maxillofacial surgery
Gibby et al,^25^ 2021	Case series	10	10	Neurosurgery
Koyachi et al,^26^ 2021	Case series	18	18	Oral and maxillofacial surgery
Ivan et al,^27^ 2021	Prospective study	11	11	Neurosurgery
Dennler et al,^28^ 2021	Case-control study	25	25	Orthopedic surgery
Liu et al,^29^ 2021	Case report	5	5	Oral and maxillofacial surgery
Scherl et al,^30^ 2021	Case report	1	1	Ear, nose, and throat surgery
Molina et al,^31^ 2021	Case report	1	1	Spine surgery
Gouveia et al,^32^ 2021	Case report	1	1	Oncologic surgery and breast oncology
Farshad et al,^33^ 2021	Case report	1	1	Spine surgery
Sugahara et al,^34^ 2021	Case report	1	1	Oral and maxillofacial surgery
Wierzbicki et al,^35^ 2022	Case report	8	8	Gastrointestinal surgery
Tang et al,^36^ 2022	Case report	7	7	Oral and maxillofacial surgery
Sasaki et al,^37^ 2022	Case report	5	5	Oral and maxillofacial surgery
Bussink et al,^38^ 2022	Case report	1	1	Oral and maxillofacial surgery
Zhou et al,^39^ 2022	Feasibility study	16	16	Neurosurgery
Yang et al,^40^ 2022	Pilot study	4	4	Oral and maxillofacial surgery
Gadodia et al,^41^ 2022	Case series	12	10	Oncologic surgery and interventional radiology
Pose-Díez-de-la-Lastra et al,^42^ 2022	Case report	1	1	Orthopedic surgery
Tokunaga et al,^43^ 2022	Case report	1	1	Gastrointestinal surgery
Lin et al,^44^ 2022	Randomized controlled trial	10	5	Oral and maxillofacial surgery
Zhou et al,^45^ 2022	Pilot study	10	10	Neurosurgery
Butler et al,^46^ 2023	Prospective case series	165	165	Spine surgery
Koyachi et al,^47^ 2023	Case report	1	1	Oral and maxillofacial surgery
Schwendner et al,^48^ 2023	Pilot study	20	20	Spine surgery
Tel et al,^49^ 2023	Retrospective observational study	5	5	Oncologic surgery and head and neck oncology
Tang et al,^50^ 2023	Pilot study	8	6	Oral and maxillofacial surgery
Rieder et al,^51^ 2024	Case report	4	4	Oral and maxillofacial surgery
Azad et al,^52^ 2024	Case-control study	50	29	Spine surgery
Javaheri et al,^53^ 2024	Case series	5	5	Gastrointestinal surgery
Ivanov et al,^54^ 2024	Case series	11	8	Oncologic surgery and pelvic organ malignancies
Guo et al,^55^ 2024	Case report	1	1	Neurosurgery
Castellarin et al,^56^ 2024	Prospective study	76	76	Orthopedic surgery
Leal et al,^57^ 2024	Case report	1	1	Orthopedic surgery
Kopriva et al,^58^ 2024	Retrospective study	97	25	Orthopedic surgery
Niloy et al,^59^ 2024	Case report	2	2	Oral and maxillofacial surgery
Altorfer et al,^60^ 2024	Case-control study	212	104	Spine surgery
Gmeiner et al,^61^ 2024	Prospective study	14	14	Neurosurgery
Kann et al,^62^ 2024	Case series	22	9	Spine surgery
Kim et al,^63^ 2024	Case report	1	1	Oral and maxillofacial surgery
Jiang et al,^64^ 2025	Case report	10	10	Ear, nose, and throat surgery
Heimann et al,^65^ 2025	Retrospective observational study	40	40	Orthopedic surgery
Kaiser et al,^66^ 2025	Case report	1	1	Neurosurgery
Huang et al,^67^ 2025	Case report/technical study	1	1	Oral and maxillofacial surgery
Gurses et al,^68^ 2025	Case report	1	1	Neurosurgery
Chang et al,^69^ 2025	Retrospective observational study	10	10	Spine surgery
Ma et al,^70^ 2025	Randomized controlled trial	150	75	Spine surgery
van Gestel et al,^71^ 2025	Prospective study	22	11	Neurosurgery
Pose-Díez-de-la-Lastra et al,^72^ 2025	Case report	1	1	Oral and maxillofacial surgery
Rojas et al,^73^ 2025	Case series	17	17	Orthopedic surgery
Lee et al,^74^ 2025	Retrospective study	34	34	Orthopedic surgery
McKenney et al,^75^ 2025	Case report	1	1	Interventional radiology
Coden et al,^76^ 2025	Retrospective control study	230	115	Orthopedic surgery
Total No. of studies	61	Total No. of cases	1547	1033

AR, augmented reality; n_AR_, number of patients included in the AR group; n_tot_, total number of patients included.

A large variety of medical applications were reported on. Oral and maxillofacial surgery (k=17, n_tot_=163, n_AR_=94), neurosurgery (k=12, n_tot_=118, n_AR_=107), spine surgery (k=11, ,n_tot_=682, n_AR_=440), and orthopedic surgery (k=10, n_tot_=552, n_AR_=335) were the most frequent medical specialties. Other specialties included oncologic surgery; gastrointestinal surgery; ear, nose, and throat (ENT) surgery; and interventional radiology.

### Quality Assessment

The majority of the studies were rated as very low certainty (k=51). This was mainly due to limitations in the sample size, study design, and lack of statistical significance. Nine studies were rated as low, and only 1 study achieved a moderate rating. No studies were rated as high certainty. A table with the full assessment results can be found in in the Supplemental Appenidx 2 (GRADE Checklist, available online at https://www.mcpdigitalhealth.org/).

### System Description

#### General Overview

Data describing the AR systems used in the included articles was extracted and categorized based on the taxonomy reported by Gsaxner et al.^15^ A full overview of these data is provided in Supplemental Appendix 3 (available online at https://www.mcpdigitalhealth.org/).

All studies targeted surgeons as the end-users and employed medical imaging as the primary data source. Most systems were designed for surgical navigation (k=34) and image-guided interventions (k=23), whereas a smaller number focused on data display (k=2).

#### Registration Paradigm and Tracking Method

Inside-out tracking was the most commonly used in the included articles (k=28), followed by outside-in tracking (k=11). An overwhelming majority used a marker-based tracking method (k=42).

#### Acquisition Time

Most navigation was based on preoperative imaging and planning data (k=45); intraoperative integration was less frequent (k=14).

#### Hardware and Software

The HoloLens (HoloLens 1 [k=11]; HoloLens 2 [k=23]) accounted for more than 65% of all studies. Other devices used were Xvision AR headset (Augmedics, k=4), NextAR Smart Glasses (Medacta, k=2), HipInsight MR headset (k=1), XR90 headset (MediView XR, k=1), HTC VIVE Pro (k=1), and Epson Moverio BT-200 (k=1). In 4 articles, the HMD was not specified.

In 18 studies, commercial platforms are used (eg, CarnaLife Holo, Opensight, SurgicalAR, Holoeyes MD, Pixee Medical, NextAR, Blueprint, Xvision, Medivis, HipInsight, ARAS, and Materialise Mimics Viewer XR). Sixteen studies reported having used their own developed software.

### Accuracy Metrics

#### Positional (Millimeters) and Angular (Degrees) Accuracy

A total of 27 studies with 186 participants reported on positional accuracy. Across all surgical use cases, the weighted mean error was 2.58 mm (range, 0.8-13.0 mm). However, all studies except 2, reporting on positional accuracy, were rated as very low certainty. The other 2 studies were rated as low certainty and reported accuracies of 2.3 mm (n_AR_=14)^16^ and 1.35 mm (n_AR_=5).^17^

Eight studies including 175 participants reported on angular accuracy. The overall weighted mean angular error was 1.26° (range, 0.59°-7.3°). Only 3 of these studies were not rated as very low certainty. These 3 studies reported angular accuracies of 1.7° (n_AR_=14),^16^ 2.4° (n_AR_=25),^18^ and 0.9° (n_AR_=40).^19^ Orthopedic surgery (k=4, n_AR_=158) dominated reporting of angular accuracies and reported a mean angular error of 1.16° (0.59°-2.5°).

Supplemental Appendix 4 shows additional accuracy results: split into specialism (Supplemental Tables C1 and C3, available online at https://www.mcpdigitalhealth.org/) and categorized per registration paradigm (Supplemental Table C2, Supplemental Figures C1 and C2, available online at https://www.mcpdigitalhealth.org/). Inside-out tracking showed the lowest weighted mean error, whereas manual registration had the highest weighted mean error.

#### Target Accuracy

In fifteen studies, clinically acceptable accuracy thresholds were explicitly defined, and all reported results met or exceeded them. These target thresholds varied by surgical specialty and were specified only for neurosurgery, oral and maxillofacial surgery, and orthopedic surgery, with no defined accuracy criteria in other fields.

• Neurosurgery: The aimed linear deviation ranged from 2.0 to 5.0 mm (k=4).^20^, ^21^, ^22^^,^^24^^,^^31^ Angular accuracy was not reported in this specialty.
• Oral and maxillofacial surgery: The aimed linear deviation ranged from 1.0 to 5.0 mm (k=8),^25^, ^26^, ^27^, ^28^, ^29^, ^30^^,^^32^, ^33^, ^34^ and the aimed angular deviation was once to be 1.0°.
• Orthopedic surgery: An aimed linear deviation was reported once (5.0 mm),^35^ and the aimed angular deviation ranged from 1.0° to 5.0° (k=3).^18^^,^^35^^,^^36^
  With the exception of the study of Kopriva et al,^58^ all these studies were rated very low certainty. Kopriva et al^58^ reported that for templated glenoid component positioning (orthopedic surgery), an angular accuracy of <5° should be achieved.

In spine surgery, accuracy was frequently assessed using the Gertzbein–Robbins grading system (GRS) for pedicle screw placement. Four studies explicitly reported target grading, with grade A or B (≤2 mm breach) considered clinically acceptable and grade A defined as optimal. In the included studies, AR-guided screw placement achieved grade A to B in 100%^37^ (very low evidence), 98.7%^38^ (low evidence), 98%^39^ (moderate evidence), and 90.5%^40^ (very low evidence) of screws placed.

#### Control Studies

Ten articles, with a total of 401 patients in the AR group, directly compared AR-guided surgery with a conventional or gold standard technique. In neurosurgery, 1 study^20^ (n_AR_=11) reported that AR guidance achieved significantly higher accuracy than free-hand procedures.

In oral and maxillofacial surgery, 3 studies with a combined total of 37 patients presented heterogeneous findings. Lin et al^44^ (n_AR_=5) showed significant improvements with AR compared with conventional optical navigation, whereas Pose-Díez-De-la-lastra et al^42^ (case report, n_AR_=1) found no difference compared with 3D-printed guides with optical tracking systems. Zhu et al^17^ (n_AR_=31) reported no significant differences between AR and surgical guides but confirmed that AR significantly outperformed free-hand techniques.

In orthopedic surgery, 2 studies evaluated 140 patients. Kopriva et al^58^ (n_AR_=25) found significant improvements in accuracy with AR over standard instrumentation, whereas Coden et al^76^ (n_AR_=115) reported no significant differences between AR and fluoroscopy-assisted total hip arthroplasty (THA).

In spine surgery, 2 studies (n_AR_=113) compared AR with robotic-assisted navigation. Altorfer et al^60^ (n_AR_=104) found that robot-assisted navigation provided significantly better accuracy than AR, whereas Kann et al^62^ (n_AR_=9) reported no significant difference between AR and robotic-assisted pedicle screw placement.

Gu et al^20^ (n_AR_=25) compared AR with conventional lumbar screw placement and reported a significant higher accuracy in the AR group. Ma et al^70^ (n_AR_=75, moderate evidence) showed significant improvement of the accuracy of pedicle screw implantation in the AR group versus conventional freehand surgery using computed tomography guidance.

### User Experience and Workflow Integration

The included studies assessed usability using heterogeneous methods. Three studies used validated tools, such as the system usability scale (SUS), the NASA Task Load Index (NASA-TLX) for workload, and the unified theory of acceptance and use of technology model combined with Likert scales. Six studies relied on study-specific questionnaires that varied substantially in scope. These questionnaires ranged from a few targeted questions to extensive surveys, such as 19- or 20-item Likert scales, and even a 58-item instrument. Four studies reported only qualitative feedback from surgeons or clinicians, without formal scoring. In contrast, 50 studies did not report any formal usability assessment and described usability only in narrative terms, such as workflow integration, ergonomics, and visualization.

### Implementation Factors

The included studies highlighted both success factors that may support the clinical adoption of AR navigation and limitations that currently restrict its widespread use. A thematic analysis identified 6 overarching themes. Success factors and limitations reported across the included studies are summarized in Table 2. Although AR navigation was consistently praised for its intuitive visualization, mobility, and educational potential, its clinical implementation remains hampered by technical, ergonomic, workflow, and regulatory challenges.

**Table 2:** Success Factors and Limitations of AR Navigation Reported in the Included Studies

Theme	Success factors	Limitations/areas for improvement	Frequency, k (%)
1. Registration and accuracy	Intuitive visual alignment; improved spatial understanding of anatomy	Suboptimal accuracy; manual registration time-consuming; no nonrigid registration; no physical safeguard against malposition	28 (45)
2. Hardware and ergonomics	Mobility and hands-free use in the OR; potential for integration without bulky external systems	HMD weight and neck strain; limited sterilizability; short battery life; refresh rate limitations	18 (29)
3. Visualization and stability	Real-time 3D overlays; clear depiction of complex anatomy (eg, nerves); reduced cognitive load	Poor contrast in bright OR light; holographic drift with head movement; dizziness, eye strain, and fatigue; visualization limited to surgeon	13 (21)
4. Workflow and usability	Potentially shorter learning curve; more intuitive than conventional navigation displays	Complex setup and calibration; workflow interruptions; imaging protocol adaptations required (eg, MRI); not feasible for emergencies; reliance on technical staff	36 (58)
5. Software and functionality	Intuitive interface; potential for markerless tracking and voice control; applications in training and planning	Tracking not robust; compatibility issues with OR environment; voice commands unreliable in noisy settings; lack of FDA/CE clearance	23 (37)
6. Evidence and validation	Potential to reduce complications and revisions; promising educational tool	Small sample sizes; single-case reports; lack of control groups and quantitative accuracy metrics; absence of outcome and cost-effectiveness data	37 (60)

AR, augmented reality; FDA, Food and Drug Administration; CE, Conformité Européenne; HMD, head-mounted display; MRI, magnetic resonance imaging; OR, operating room.

### Operational Metrics

#### Time Impact

Across the included studies, findings on operative time were mixed. A number of studies reported increases in operative duration with AR, including work in ENT, gastrointestinal, neurosurgical, oncologic, oral and maxillofacial, orthopedic, and spine surgeries. Reported increases ranged from a few minutes up to approximately 20 minutes, depending on the procedure and AR workflow.

Conversely, several studies reported decreases in operative time, including work in gastrointestinal, neurosurgical, oral and maxillofacial, and spine surgeries. These reductions were generally procedure or task specific and varied from modest improvements to substantial time savings. A smaller number of studies found no clear difference in operative duration, most commonly in oncologic and orthopedic surgeries.

#### Radiation Exposure

Radiation outcomes were reported in neurosurgery, orthopedic surgery, and spine surgery. Moreover, AR consistently reduced fluoroscopy use, such as in neurosurgery (1.2 vs 4 minutes^22^), orthopedics (a decrease of 37% radiation dose and 50% of fluoroscopy time^42^; no fluoroscopy required^45^), and spine surgery (less fluoroscopy used^44^; fully fluoroscopy-free, except in S1 screw placement^46^).

## Discussion

### Summary of Findings

This systematic review evaluated the current state of clinically tested HMD-AR navigation systems in surgery. Primary outcomes were (1) accuracy and (2) usability in clinical workflows, including success factors and barriers to implementation. Secondary outcomes included technical system characteristics (hardware, tracking, and registration methods), comparative performance against conventional techniques, required preparation time, and their impact on operative time and radiation exposure.

### Study Characteristics and Quality Assessment

This review highlights the growing interest and application of AR in surgical practice, with a notable increase in publications throughout the inclusion period (2018-2025). However, the dominance of case reports and series indicates the exploratory nature of AR integration in clinical settings, whereas the limited number of only 2 randomized controlled trials reflects a gap in high-level evidence. Further support for this comes from the GRADE evaluation. Only 1 study^39^ reached a moderate certainty rating. The study was in the field of spine surgery, which may reflect the relative maturity of surgical navigation in spinal procedures, where validated metrics for accuracy (eg, GRS) and established workflows facilitate more robust study designs and outcome assessments.

### System Characteristics

Clinical studies demonstrate that AR has been applied across a wide range of surgical specialties, with oral and maxillofacial surgery, neurosurgery, spine surgery, and orthopedic surgery being the most frequently represented. More than 65% of the included studies used HoloLens devices, with marker-based inside-out tracking as the most used method. The HoloLens is relatively low-cost, widely available, and easy to use, which makes it attractive for clinical and research applications. Its accessibility may support AR adoption but could also limit innovation by discouraging exploration of alternative systems that might offer better accuracy or integration. With Microsoft ending support for HoloLens 2 after 2027 and no successor announced, concerns about long-term sustainability are growing. AR software should be adaptable to evolving hardware platforms, allowing integration with future device improvements and innovations.

### Accuracy

Although many studies report sub–3-mm accuracy, these findings predominantly stem from very low certainty evidence. Therefore, they should be interpreted as proof-of-concept results. Although these accuracy values broadly overlap with the accuracy typically achieved by established surgical navigation modalities,^77^, ^78^, ^79^ their interpretation is limited by heterogeneous methods, small samples, and inconsistent accuracy definitions. This constrain cross-study comparability and high level evidence. Furthermore, substantial variation in accuracy across studies underscores the influence of system configuration, registration method, and anatomy. For example, studies using older hardware generations or manual registration consistently reported higher errors. This is seen in ENT surgery,^51^^,^^52^ where notable higher inaccuracies were observed.

AR systems consistently met or exceeded defined target accuracy thresholds. However, these were reported only for neurosurgery (2-5 mm), oral and maxillofacial surgery (1-5 mm), and spine surgery. For spine procedures, the GRS is commonly used to evaluate pedicle screw accuracy, where grade A to B (<2 mm cortical breach) are considered clinically acceptable. In a large study by Katsumi et al^80^ on intraoperative computed tomography–based navigation (without AR)^53^ 97.1% of 1082 pedicle screws fell within this clinically acceptable range (grade A: 93.3%; grade B: 3.8%). In comparison, 3 of 4 AR-guided cohorts in the present study achieved similar or superior GRS performance (moderate to very low certainty levels), whereas 1 cohort showed lower occurrence (90.5%) of GRS grade A or B (very low certainty level). It is important to note that this grading system only reflects whether a screw remains within the pedicle/bone, not how closely it follows the planned trajectory. In cases with wide pedicles, substantial deviations from the planned path can still score as grade A, making the metric anatomy dependent. In studies including a control group, AR consistently outperformed free-hand methods but showed mixed results against other modalities such as robotic or optical navigation.

### User Experience, Usability, and Implementation factors

Usability assessments were inconsistent: only 3 studies used validated tools (eg, SUS, NASA-TLX, unified theory of acceptance and use of technology), whereas most relied on custom surveys or qualitative feedback. More than 80% of studies lacked formal usability evaluation, limiting comparability across studies. Despite limited formal evaluation, AR was often praised for intuitive visualization and workflow compatibility, although concerns about ergonomics and calibration were noted.

Six key factors influencing adoption were identified. Success factors included intuitive interfaces and educational value; limitations involved technical reliability, headset comfort, workflow integration, and regulatory challenges. Limited formal usability evaluation further complicates adoption and standardization across clinical settings. Importantly, user experience and usability are highly dependent on the specific software implementation, hardware platform, registration strategy, and tracking method used. Given the limited number of studies, these factors act as potential confounders and should ideally be evaluated separately to enable meaningful comparison across systems.

### Time Impact and Radiation Exposure

Overall, evidence on time impact was low and did not point to a consistent effect of AR on operative time; rather, outcomes varied both between and within specialties. This inconsistency suggests that operative duration is strongly influenced by factors such as workflow integration, surgeon experience, and the specific AR system used. Compared with surgical time findings, evidence supporting reduced fluoroscopy and radiation exposure was more consistent across specialties, indicating a clearer benefit of AR in this domain.

### Comparison With Previous Studies

Several systematic reviews have examined AR in surgery but differ in scope and maturity of evidence. Tu et al^81^ examined how HMDs can enhance surgical context-aware systems in open surgery, highlighting potential gains in situational awareness and decision making; however, most included studies were preclinical or early stage (phantom, cadaver, or simulation). This review identified recurring constraints such as latency, limited field of view, data heterogeneity, and occlusion from egocentric views.

Asadi et al^82^ provided a broader intraoperative AR overview spanning multiple hardware categories (HMDs, microscopes, tablets, and projectors) and surgical domains. This review reported improvements in accuracy (eg, pedicle screws), operative time, blood loss, and radiation exposure in selected large clinical series, whereas noting persistent challenges: learning curves, hardware limitations (field of view, resolution, and battery life), workflow complexity (setup or registration), sparse real-time or soft-tissue tracking, and a lack of standardized usability metrics.

Malhotra et al^83^ focused on the methodological landscape of AR evaluations and reported that despite rapid technical advances in AR since 2008, evaluation and validation practices remain fragmented, inconsistent, and often inadequate. They proposed organizing evaluation across 4 interdependent domains to capture the multidimensional nature of AR performance: user-, procedure-, system-, and patient-related metrics.

Our findings complement earlier reviews by providing a focused, clinically grounded overview of HMD-based navigation systems. By including only studies conducted in real surgical settings, this review offers a comprehensive summary of clinical applications across surgical domains, detailing the types of systems used, their intended use cases, and reported accuracy levels. Moreover, it identifies the performance thresholds and practical requirements necessary for successful clinical implementation, thereby offering a clearer understanding of both the opportunities and the challenges of AR integration in surgery.

### Recommendations

Given the substantial heterogeneity observed in system configurations, study designs, and reporting practices, future research must adopt more standardized evaluation methods to enable meaningful comparison across studies and accelerate clinical translation. On the basis of the findings in this review and supported by recent methodological reviews, the following minimum reporting set for future AR surgical navigation studies is suggested.

• Usability: use validated tools, such as SUS questionnaire to quantify usability instead of relying on descriptive impressions.
• Workload: include raw NASA-TLX to capture cognitive and physical workload demands.
• Ergonomics: capture headset comfort or fit, visual fatigue, and cybersickness. Ergonomic burden and visual strain are recurrent barriers to clinical adoption of HMDs; structured reporting makes results comparable. A validated tool should be created.
• Technical accuracy: report the accuracy of the AR system using standardized measures (eg, target registration error and fiducial registration error), with SD and a registration robustness ratio (successful registrations or attempts). Additionally, a transparent method for the measurements needs to be provided. Use standard definitions from image-guided surgery, and where possible benchmark against the clinical gold standard for the indication or predefined, specialty-specific thresholds informed by current surgical navigation performance.
• Workflow impact: provide objective measures including setup and registration time, frequency of AR-related workflow interruptions, and re-registration count.
• System stability: report network/calibration errors, test intraoperative registration errors multiple times, or other indicators of overall system reliability.
• Patient-level outcomes: include at minimum intraoperative safety events and operative time.

## Conclusion

Although AR navigation demonstrates encouraging technical performance and potential reductions in radiation exposure, the underlying evidence is predominantly of very low certainty, and any impression of pooled robustness should be avoided. Current clinical studies remain preliminary and largely focus on feasibility and technical performance rather than patient-centered outcomes. Robust evidence from larger, controlled trials is still required to assess clinical benefit before AR navigation can be considered for routine surgical adoption. Furthermore, clinical integration continues to be hindered by technical-, ergonomic-, and workflow-related barriers.

To move toward clinical implementation, future evaluations should compare new AR systems to the current clinical gold standard for their intended use. To support superiority assessments and draw robust conclusions regarding clinical benefit, larger sample sizes will be necessary. Furthermore, validated usability questionnaires and standardized clinical and technical performance metrics with specialty-specific thresholds should be used. In the Netherlands, initiatives are ongoing to establish joint consensus through expert discussions (first, nationally, and then internationally) to develop these standards. Such guidelines should be applied to ensure systems meet predefined requirements and to enable meaningful comparison across studies. This approach will accelerate implementation, prevent repeated reinvention within research groups, and ensure the safe integration of AR into clinical practice. Collaboration among clinicians, engineers, and regulators will be key to transforming AR from experimental technology into a reliable, sustainable clinical tool. System design should prioritize alignment with clinical workflows and the practical needs of surgeons.

## Potential Competing Interests

The authors report no competing interests.

## Supplemental Online Material

Supplementary Material

Supplementary Checklist
