# Supplementary material for: Augmented Reality in Navigated Surgery: A Systematic Review of Clinical Accuracy and System Performance
Source: Mayo Clin Proc Digit Health. 2026 Apr 4;4(2):100358. doi: 10.1016/j.mcpdig.2026.100358 (PMC13144587; doi:10.1016/j.mcpdig.2026.100358)
Supplement: Supplementary Material [file mmc1.pdf]

Article	Initial GRADE	Final CoE (GRADE)
Incekara et al.	Low	Very low
Zhu et al.	Low	Very low
Maruyama et al.	Low	Very Low
Gregory et al.	Low	Very Low
Gu et al.	Low	Very Low
Yao et al.	Low	Very Low
Scherl et al.	Low	Very Low
Molina et al.	Low	Very Low
Sun et al.	Low	Very Low
Gibby et al.	Low	Very Low
Koyachi et al.	Low	Very Low
Ivan et al.	Low	Very Low
Dennler et al.	Low	Very Low
Liu et al.	Low	Very Low
Scherl et al.	Low	Very Low
Molina et al.	Low	Very Low
Gouveia et al.	Low	Very Low
Farshad et al.	Low	Very Low
Sugahara et al.	Low	Very Low
Wierzbicki et al.	Low	Very Low
Tang et al.	Low	Very Low
Sasaki et al.	Low	Very Low
Bussink et al.	Low	Very Low
Zhou et al.	Low	Very Low
Yang et al.	Low	Very Low
Gadodia et al.	Low	Very Low
Pose-Díez-de-la-	Low	Very Low
Tokunaga et al.	Low	Very Low
Lin et al.	High	Low
Zhou et al.	Low	Very Low
Butler et al.	Low	Very Low
Koyachi et al.	Low	Very Low
Schwendner et	Low	Very Low
Tel et al.	Low	Very Low
Tang et al.	Low	Very Low
Rieder et al.	Low	Very Low
Azad et al.	Low	Very Low
Javaheri et al.	Low	Very Low
Ivanov et al.	Low	Very Low
Guo et al.	Low	Very Low
Castellarin et	Low	Very Low
Leal et al.	Low	Very Low
Kopriva et al.	Low	Low
Niloy et al.	Low	Very Low

Altorfer et al.	Low	Low
Gmeiner et al.	Low	Low
Kann et al.	Low	Very Low
Kim et al.	Low	Very Low
Dongwen et al.	Low	Very Low
Heimann et al.	Low	Low
Kaiser et al.	Low	Very Low
Huang et al.	Low	Very Low
Gurses et al.	Low	Very Low
Chang et al.	Low	Low
Ma et al.	High	Moderate
van Gestel et al.	Low	Low
Pose-Díez-de-la-	Low	Very Low
Rojas et al.	Low	Very Low
Lee et al.	Low	Low
McKenney et al.	Low	Very Low
Coden et al.	Low	Low
	Initial GRADE	Final CoE (GRADE)
Very Low	0	51
Low	59	9
Moderate	0	1
High	2	0
