# Supplementary material for: Augmented Reality in Navigated Surgery: A Systematic Review of Clinical Accuracy and System Performance
Source: Mayo Clin Proc Digit Health. 2026 Apr 4;4(2):100358. doi: 10.1016/j.mcpdig.2026.100358 (PMC13144587; doi:10.1016/j.mcpdig.2026.100358)
Supplement: Supplementary Appendix A [file mmc3.docx]

**Appendix A Query strings**

**Filters:**

*From 2018-2025*

**Pubmed**

#1 "Augmented Reality"[Mesh] OR "Augmented Realit*"[tiab] OR "Mixed Realit*"[tiab] OR "hybrid realit*"[tiab] OR "head mounted display"[tiab] OR "heads up display*"[tiab] OR "immersive technolog*"[tiab] OR "extended realit*"[tiab] OR "enlarged realit*"[tiab] OR (Realit*[tiab] AND (Augmented[tiab] OR Mixed[tiab] OR Extended[tiab]))

#2 "Surgery, Computer-Assisted"[Mesh] "Computer-Assisted Surger*"[tiab] OR "Computer Assisted Surger*"[tiab] OR "CAS"[tiab] OR "CSN"[tiab] OR "Computer-Assisted Intervention*"[tiab] OR "Computer-Aided Surger*"[tiab] OR "Computer Aided Surger*"[tiab] OR "Image Guided Surger*"[tiab] OR "Image-Guided Surger*"[tiab] OR "IGS"[tiab] OR "Surgical Navigation"[tiab]

**Embase**

#1 'augmented reality'/exp OR 'augmented realit*':ti,ab OR 'mixed realit*':ti,ab OR 'hybrid realit*':ti,ab OR 'head mounted display*':ti,ab OR 'heads up display*':ti,ab OR 'immersive technolog*':ti,ab OR 'extended realit*':ti,ab OR 'enlarged realit*':ti,ab OR (realit*:ti,ab AND (augmented:ti,ab OR mixed:ti,ab OR extended:ti,ab))

#2 'computer assisted surgery'/exp OR 'computer-assisted surger*':ti,ab OR 'computer assisted surger*':ti,ab OR 'cas':ti,ab OR 'csn':ti,ab OR 'computer-assisted intervention*':ti,ab OR 'computer-aided surger*':ti,ab OR 'computer aided surger*':ti,ab OR 'image guided surger*':ti,ab OR 'image-guided surger*':ti,ab OR 'igs':ti,ab OR 'surgical navigation':ti,ab

#3 1 and 2

**Scopus**

#1 ( "augmented realit*" OR "mixed realit*" OR "hybrid realit*" OR "head mounted display*" OR "heads up display*" OR "immersive technolog*" OR "extended realit*" OR "enlarged realit*" OR ( realit* AND ( augmented OR mixed OR extended ) ) )

#2 ( "computer-assisted surger*" OR "computer assisted surger*" OR "CAS" OR "CSN" OR "computer-assisted intervention*" OR "computer-aided surger*" OR "computer aided surger*" OR "image guided surger*" OR "image-guided surger*" OR "IGS" OR "surgical navigation" )

#3 #1 AND #2

**Web of Science Core Collection**

#1 TS=("augmented realit*" OR "mixed realit*" OR "hybrid realit*" OR "head mounted display*" OR "heads up display*" OR "immersive technolog*" OR "extended realit*" OR "enlarged realit*" OR (realit* AND (augmented OR mixed OR extended)))

#2 TS=("computer-assisted surger*" OR "computer assisted surger*" OR "CAS" OR "CSN" OR "computer-assisted intervention*" OR "computer-aided surger*" OR "computer aided surger*" OR "image guided surger*" OR "image-guided surger*" OR "IGS" OR "surgical navigation")
