# Supplementary material for: Augmented Reality in Navigated Surgery: A Systematic Review of Clinical Accuracy and System Performance
Source: Mayo Clin Proc Digit Health. 2026 Apr 4;4(2):100358. doi: 10.1016/j.mcpdig.2026.100358 (PMC13144587; doi:10.1016/j.mcpdig.2026.100358)
Supplement: Supplementary Appendix B [file mmc4.docx]

**Appendix B AR System description table**

| **Author** | **Publication year** | **User case** | **Application** | **Registration paradigm** | **Tracking method** | **Aquisition time** | **Hardware device** | **Software** |
| --- | --- | --- | --- | --- | --- | --- | --- | --- |
| Incekara, F., et al. | 2018 | Neurosurgery | Image-guided intervention | Manual | NA | Pre-interventional | HoloLens 1 | ITK-SNAP + Meshmixer + VertoStudio |
| Maruyama, et al. | 2018 | Neurosurgery | Image-guided intervention | Inside-out | Marker-based | Pre-interventional | Epson Moverio BT-200 smart glasses (Seiko Epson Corp) | Software created in Unity |
| Zhu, M., et al. | 2018 | Oral and Maxillofacial Surgery | Image-guided intervention | Inside-out | Marker-based | Pre-interventional | - | AR Toolkit |
| Gregory, T. M. et al. | 2018 | Orthopedic Surgery | Image-guided intervention | Manual | NA | Pre-interventional | HoloLens | TeraRecon Holoportal |
| Scherl et al. | 2020 | Ear, Nose, and Throat Surgery | Image-guided intervention | Manual | NA | Pre-interventional | HoloLens 1 | - |
| Yao et al. | 2020 | Neurosurgery | Surgical navigation | Inside-out | Marker-based | Both |  | HuaxiAR |
| Gibby, J., et al. | 2020 | Neurosurgery | Surgical navigation | Inside-out | Marker-based | Pre-interventional | HoloLens | OpenSight AR (Navarad) |
| Sun et al. | 2020 | Oral and Maxillofacial Surgery | Surgical navigation | Outside-in | Marker-based | Pre-interventional | HoloLens | Custom AR surgical navigation system with TCP/IP communication |
| Gu et al | 2020 | Spine Surgery | - | - | - | Both | HoloLens 2 | - |
| Molina, C.A., et al. | 2020 | Spine Surgery | Surgical navigation | Inside-out | Marker-based | Both | xvision (Augmedics) | Proprietary platform with MITK-based analysis |
| Scherl et al. | 2021 | Ear, Nose, and Throat Surgery | Image-guided intervention | Manual | NA | Pre-interventional | HoloLens 1 | - |
| Ivan et al. | 2021 | Neurosurgery | Image-guided intervention | Manual | Marker-les | Pre-interventional | HoloLens | Opensight |
| Liu et al. | 2021 | Oral and Maxillofacial Surgery | Surgical navigation | Outside-in | Marker-based | Both | HoloLens 1 | - |
| Gouveia, P.F., et al. | 2021 | Oncologic Surgery | Image-guided intervention | Inside-out | Marker-based | Pre-interventional | HoloLens 1 | - |
| Koyachi et al. | 2021 | Oral and Maxillofacial Surgery | Surgical navigation | Inside-out | Marker-based | Pre-interventional | HoloLens 1 | - |
| Sugahara, K. et al. | 2021 | Oral and Maxillofacial Surgery | Image-guided intervention | Manual | NA | Pre-interventional | HoloLens | Custom Unity-based application using CT segmentation and 3D modeling |
| Dennler at al. | 2021 | Orthopedic Surgery | Data display | NA | NA | Pre-interventional | HoloLens 1 | - |
| Molina et al. | 2021 | Spine Surgery | Surgical navigation | Inside-out | Marker-based | Both | xvision | - |
| Farshad et al. | 2021 | Spine Surgery | Surgical navigation | Inside-out | Marker-based | Pre-interventional | HoloLens 2 | Custom AR navigation system using Aruco marker tracking and in-house planning software |
| Wierzbicki et al. | 2022 | Gastrointestinal Surgery | Data display | NA | NA | NA | HoloLens 2 | CarnaLife Holo |
| Tokunaga et al. | 2022 | Gastrointestinal Surgery | Image-guided intervention | Manual | Marker-less | Pre-interventional | HoloLens 1 | SYNAPSE VINCENT + Holoeyes MD |
| Zhou et al. | 2022 | Neurosurgery | Surgical navigation | Inside-out | NA | Pre-interventional | HoloLens 2 | - |
| Zhou, Z. et al | 2022 | Neurosurgery | Surgical navigation | Inside-out | Marker-based | Pre-interventional | HoloLens 2 | Custom mixed reality navigation system (developed at Tianjin University) |
| Gadodia, G., et al. | 2022 | Oncologic Surgery | Image-guided intervention | Outside-in | Marker-based | Both | HoloLens 1 | - |
| Tang et al. | 2022 | Oral and Maxillofacial Surgery | Surgical navigation | Outside-in | Marker-based | Pre-interventional | HoloLens 1 / 2 and other | - |
| Sasaki et al. | 2022 | Oral and Maxillofacial Surgery | Image-guided intervention | Manual | NA | Pre-interventional | HoloLens 2 | - |
| Bussink et al. | 2022 | Oral and Maxillofacial Surgery | Surgical navigation | Inside-out | Marker-based | Pre-interventional | HoloLens 2 | - |
| Yang, R. | 2022 | Oral and Maxillofacial Surgery | Surgical navigation | Outside-in | Marker-based | Pre-interventional | Microsoft Hololens | Custom-built using Unity3D, Mixed Reality Toolkit, IGSTK, QT |
| Lin, L., et al. | 2022 | Oral and Maxillofacial Surgery | Surgical navigation | Inside-out | Marker-based | Pre-interventional | HoloLens | software developed in Unity |
| Pose-Díez-de-la-Lastra, A. et al. | 2022 | Orthopedic Surgery | Image-guided intervention | Inside-out | Marker-based | Pre-interventional | HoloLens 2 | Custom Unity-based applications using Vuforia SDK and MRTK |
| Tel, A., et al. | 2023 | Oncologic Surgery | Image-guided intervention | Manual | NA | Pre-interventional | HoloLens 2 & iPad | Materialise Mimics Viewer |
| Koyachi, M., et al. | 2023 | Oral and Maxillofacial Surgery | Image-guided intervention | Inside-out | Marker-based | Pre-interventional | HololLens 2 | Holoeyes MD (Holoeyes, Tokyo, Japan) application |
| Tang, et al. | 2023 | Oral and Maxillofacial Surgery | Surgical navigation | Outside-in | Marker-based | Pre-interventional | HoloLens 2 | Unity-based mixed reality interface |
| Butler et al. | 2023 | Spine Surgery | Surgical navigation | Inside-out | Marker-based | Both | xvision | - |
| Schwendner, M., et al. | 2023 | Spine Surgery | Surgical navigation | Outside-in | Marker-based | Intra-interventional | NextAR Smart Glasses (Medacta) | NextAR TS navigation system |
| Javaheri et al | 2024 | Gastrointestinal Surgery | Image-guided intervention | Inside-out | Marker-based | Both | HoloLens 2 | ARAS |
| Guo et al. | 2024 | Neurosurgery | Surgical navigation | Outside-in | Marker-based | Pre-interventional | HoloLens 2 | Medivis SurgicalAR system |
| Gmeiner, M., et al. | 2024 | Neurosurgery | Surgical navigation | Outside-in | Marker-based | Pre-interventional | HoloLens 2 | CORTEXPLORER MED (cortEXplore GmbH) |
| Ivanov, V.M. et al. | 2024 | Oncologic Surgery | Surgical navigation | Inside-out | Marker-based | Pre-interventional | HoloLens 2 | Custom AR software + 3D Slicer 5.2 (HaSC-AR-v1) |
| Rieder et al. | 2024 | Oral and Maxillofacial Surgery | Surgical navigation | Inside-out | Marker-les | Pre-interventional | HoloLens 2 | - |
| Niloy, I. et al. | 2024 | Oral and Maxillofacial Surgery | Image-guided intervention | Outside-in | Marker-based | Pre-interventional | HoloLens | Custom surgical planning and navigation platform |
| Kim H. J. et al. | 2024 | Oral and Maxillofacial Surgery | Image-guided intervention | Inside-out | Marker-based | Pre-interventional | VIVE pro, HTC, Taoyuan, Taiwan | In house developed VR simulation software using Unity 3D |
| Castellarin et al. | 2024 | Orthopedic Surgery | Surgical navigation | Inside-out | Marker-based | Pre-interventional | Smart glasses + tablet (Pixee Medical Knee+ AR) | Pixee Medical AR navigation system |
| Leal, J., et al. | 2024 | Orthopedic Surgery | Image-guided intervention | Inside-out | Marker-based | Pre-interventional | HipInsight mixed reality headset (Surgical Planning Associated, Inc.) | Proprietary surgical planning and navigation platform |
| Kopriva, J.M. et al. | 2024 | Orthopedic Surgery | Image-guided intervention | Inside-out | Marker-less | Pre-interventional | HoloLens 2 | Blueprint planning software + MR visualization |
| Azad et al. | 2024 | Spine Surgery | Surgical navigation | Inside-out | Marker-based | Both | xvision | Xvision |
| Altorfer, F.C.S., et al | 2024 | Spine Surgery | Surgical navigation | Inside-out | Marker-based |  | Xvision AR headset (Augmedics) | Proprietary navigation platform |
| Kann, M.R | 2024 | Spine Surgery | Surgical navigation | Inside-out | Marker-based | Intra-interventional | xvision AR-HMD (Augmedics, Chicago, IL, USA) | Integrated AR navigation software by Augmedics |
| Dongwen et al. | 2025 | Ear, Nose, and Throat Surgery | Surgical navigation | Inside-out | Marker-based | Pre-interventional | HoloLens 2 | HoloLens Ear Image Guidance |
| McKenney A. S. et al. | 2025 | Interventional Radiology | Surgical navigation | Outside-in | Marker-based | Intra-interventional | XR90 headset (MediView XR, Inc.) | magineHIVE (customized 3D Slicer + TotalSegmentator) |
| Kaiser et al. | 2025 | Neurosurgery | Image-guided intervention | Inside-out | Marker-based | Pre-interventional | - | - |
| Gurses et al. | 2025 | Neurosurgery | Image-guided intervention | Inside-out | - | Pre-interventional | HoloLens 2 | SurgicalAR |
| van Gestel et al. | 2025 | Neurosurgery | Surgical navigation | Inside-out | Marker-based | Pre-interventional | HoloLens 2 | Custom AR navigation system (in-house developed) |
| Huang et al. | 2025 | Oral and Maxillofacial Surgery | Surgical navigation | Inside-out | Marker-based | Pre-interventional | HoloLens 2 | - |
| Pose-Díez-de-la-Lastra, A. et al. | 2025 | Oral and Maxillofacial Surgery | Surgical navigation | Inside-out | Marker-based | Pre-interventional | HoloLens 2 | Custom Unity-based applications using Vuforia SDK and MRTK |
| Heimann et al. | 2025 | Orthopedic Surgery | Surgical navigation | Inside-out | Marker-based | Pre-interventional | HoloLens 2 | SPQ Registration Application |
| Rojas, J.T., et al. | 2025 | Orthopedic Surgery | Surgical navigation | Outside-in | Marker-based | Pre-interventional | NextAR shoulder system (Medacta) + HMD | Proprietary planning and navigation platform |
| Lee, M. Y. et al. | 2025 | Orthopedic Surgery | - | - | Marker-based | Pre-interventional | AR-headset (- which one) | - |
| Coden, G., et al. | 2025 | Orthopedic Surgery | Image-guided intervention | - | NA | Pre-interventional | HoloLens | - |
| Chang et al. | 2025 | Spine Surgery | Surgical navigation | Outside-in | Marker-based | Both | - | - |
| Ma et al. | 2025 | Spine Surgery | Surgical navigation | Outside-in | Marker-based | Both | HoloLens 2 | - |
