# Supplementary material for: Augmented Reality in Navigated Surgery: A Systematic Review of Clinical Accuracy and System Performance
Source: Mayo Clin Proc Digit Health. 2026 Apr 4;4(2):100358. doi: 10.1016/j.mcpdig.2026.100358 (PMC13144587; doi:10.1016/j.mcpdig.2026.100358)
Supplement: Supplementary Appendix C [file mmc5.docx]

**Appendix C Additional analysis accuracy results**

*Table C.1. Reported positional (mm) accuracy. Only specialism where the accuracy was reported are included in the Table.*

| **Use case** | **k (# of studies)** | **n_AR_ (# of patients in AR group)** | **Weighted mean (mm)** | **Min–Max (mm)** |
| --- | --- | --- | --- | --- |
| Ear, Nose, and Throat Surgery | 2 | 8 | 12.48 | 12.4 – 13.0 |
| Neurosurgery | 8 | 83 | 2.56 | 1.5 – 4.0 |
| Oncologic Surgery | 1 | 10 | 1.2 | N/A |
| Oral & Maxillofacial Surgery | 11 | 60 | 1.78 | 0.8 – 5.5 |
| Orthopedic Surgery | 1 | 17 | 2.00 | N/A |
| Spine Surgery | 2 | 2 | 2.79 | 2.1 - 3.5 |
| **Total** | **26** | **186** | **2.58** | **0.8** **- 13.0** |

*Table C.2.. Reported positional (mm) accuracy categorized by registration paradigm. Only specialism where the accuracy was reported are included in the Table.*

| **Outside-in** |  |  |  |
| --- | --- | --- | --- |
| **Use case** | **k** | **n_AR_** | **Weighted mean (mm)** |
| Oral and Maxillofacial Surgery | 6 | 25 | 2.58 |
| Orthopedics | 1 | 17 | 2.00 |
| Neurosurgery | 1 | 14 | 2.30 |
| Oncologic Surgery | 1 | 10 | 1.20 |
| All of the above | 9 | 66 | 2.16 |
| **Inside-out** |  |  |  |
| **Use case** | **k** | **n_AR_** | **Weighted mean (mm)** |
| Oral and Maxillofacial Surgery | 6 | 40 | 1.24 |
| Neurosurgery | 6 | 44 | 1.82 |
| Spine Surgery | 1 | 1 | 2.07 |
| All of the above | 13 | 85 | 1.55 |
| **Manual** |  |  |  |
| **Use case** | **k** | **n_AR_** | **Weighted mean (mm)** |
| Ear, Nose, and Throat Surgery | 2 | 8 | 12.48 |
| Neurosurgery | 1 | 25 | 4.00 |
| All of the above | 3 | 33 | 6.05 |

*Table C.3. Reported angular (°) accuracy. Only specialism where the accuracy was reported are included in the table.*

| **Specialism** | **k (# of studies)** | **n_AR_ (# of patients in AR group)** | **Weighted mean (°)** | **Min–Max (°)** |
| --- | --- | --- | --- | --- |
| Neurosurgery | 1 | 14 | 1.71 | N/A |
| Oral & Maxillofacial Surgery | 1 | 1 | 0.80 | N/A |
| Orthopedic Surgery | 4 | 158 | 1.16 | 0.6 - 2.5 |
| Spine Surgery | 2 | 2 | 4.86 | 2.4 - 7.3 |
| **Total** | **8** | **175** | **1.26** | **0.6 - 7.3** |


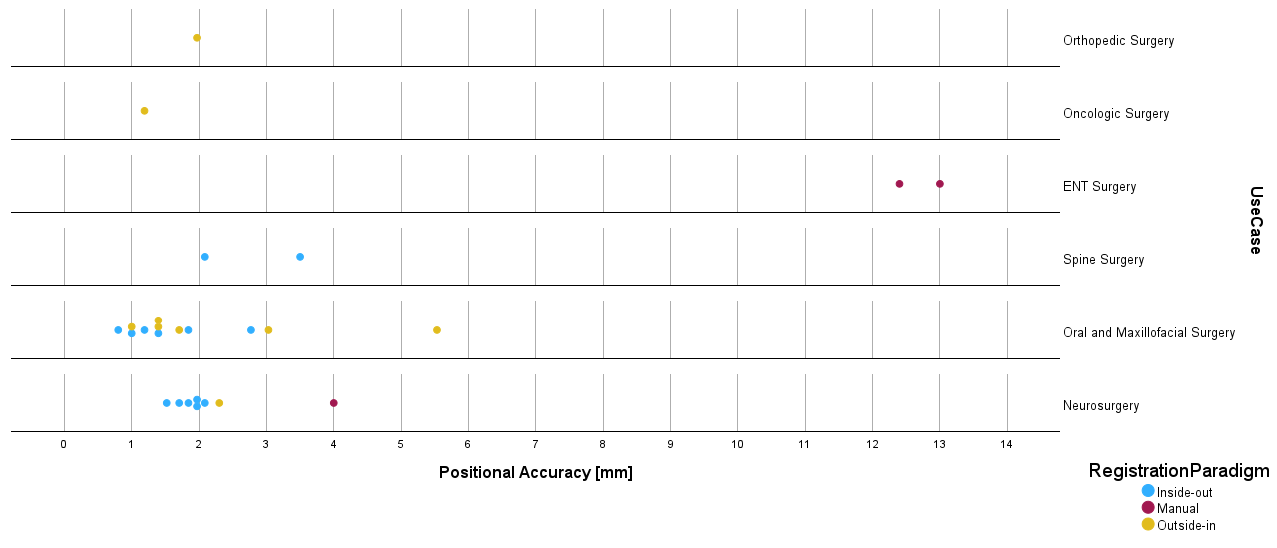


Figure C.1. Reported *positional* (mm) accuracy categorized by registration paradigm. Only specialism where the accuracy was reported are included in the figure.


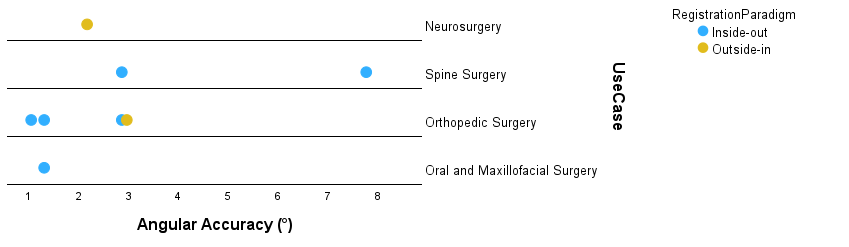


Figure C.2. Reported angular (°) accuracy categorized by registration paradigm. Only specialism where the accuracy was reported are included in the figure.
